# Does the Bethesda category predict aggressive features in differentiated thyroid cancer?

**DOI:** 10.20945/2359-3997000000098

**Published:** 2019-02-01

**Authors:** Ana Rafaela Lopes Reis Lima, Karoline Matias Morais de Medeiros, Conceição de Maria Ribeiro Veiga Parente, Adriana de Sá Caldas, Manuel dos Santos Faria, Marcelo Magalhães, Carla Souza Pereira Sobral

**Affiliations:** 1 Universidade Federal do Maranhão Universidade Federal do Maranhão Hospital Universitário Serviço de Endocrinologia São Luís MA Brasil Serviço de Endocrinologia do Hospital Universitário da Universidade Federal do Maranhão (UFMA), São Luís, MA, Brasil; 2 Universidade Federal do Maranhão Universidade Federal do Maranhão Centro de Pesquisas Clínicas São Luís MA Brasil Centro de Pesquisas Clínicas, Universidade Federal do Maranhão (UFMA), São Luís, MA, Brasil

**Keywords:** Bethesda, thyroid, cancer, aggressiveness

## Abstract

**Objective:**

To analyze the importance of preoperative cytology of thyroid nodules and its relationship with mortality risk, recurrence risk, dynamic stratification, and aggressive characteristics (vascular invasion, aggressive histology, incomplete tumor resection, extrathyroidal extension of the tumor, and presence of lymph node and distant metastases).

**Subjects and methods:**

Retrospective evaluation of 153 patients diagnosed with differentiated thyroid carcinoma (DTC) and following up at the *Hospital Universitário Presidente Dutra* between January 1999 and December 2016.

**Results:**

In all, 96% of the patients were female, 79.7% had papillary carcinoma and the most common fine-needle aspiration (FNA) result was Bethesda II (29.4%). The mean age was 43.11 ± 12.8 years. Overall, 85% of the patients progressed without any evidence of disease. There was a statistically significant relationship between the presurgical FNA and the presence of extrathyroidal extension, vascular invasion, and lymph node metastasis.

**Conclusions:**

The preoperative cytology of the nodule may have an impact on the follow-up of patients with DTC. Future studies in a larger population are required to confirm this finding.

## INTRODUCTION

Differentiated thyroid cancer (DTC) arises from a normal follicular thyroid cell and is responsible for most (> 90%) thyroid carcinomas (1). Papillary thyroid carcinoma (PTC) is the most frequent DTC (affecting 95% of all cases), followed by follicular carcinoma, Hürthle cell carcinoma, and poorly DTC (1,2).

After surgical removal and histopathological confirmation of thyroid cancer, the patients are classified according to their mortality risk (MR) and recurrence risk (RR). These two classification systems help physicians evaluate the surgical result and decide about the initial treatment and follow-up of the patient (2). These systems also facilitate the communication among health care professionals.

The MR is evaluated using the American Joint Committee on Cancer/International Union Against Cancer (AJCC/UICC) tumor-node-metastasis (TNM) staging system, which is based on a combination of age at diagnosis, size of the primary tumor, extrathyroidal tumor extension, and the presence of lymph node and distant metastases (2,3). Since this system fails to predict the RR, we use instead the RR system, which analyzes features such as vascular invasion, aggressive histology, tumor resection, and postoperative thyroglobulin level (3–5).

After the initial treatment, a dynamic stratification system is used to classify the treatment response according to clinical, biochemical, and imaging (structural and functional) data obtained during follow-up. According to this system, the patients are categorized as having an excellent response, incomplete biochemical response, indeterminate response, or incomplete structural response (2,3).

These classification systems adequately define the best initial follow-up strategy for each patient (requirement of additional surgery, radioiodine therapy, and/or hormone suppressive therapy) and prevent overtreatment. However, these systems do not take into account preoperative characteristics like the cytological features of the nodule. This information may be important for adequate surveillance. Based on that, this study analyzed the importance of preoperative cytological analysis of thyroid nodules, obtained by ultrasound-guided fine-needle aspiration (FNA), and the relationship of preoperative cytological features of the nodule with the patients’ MR, RR, and dynamic stratification, and the tumor's aggressive characteristics (vascular invasion, aggressive histology, incomplete tumor resection, extrathyroidal extension of the tumor, and presence of lymph node/distant metastases).

## SUBJECTS AND METHODS

This retrospective study analyzed the records of 505 patients with DTC seen at the *Hospital Universitário Presidente Dutra* in São Luís (Maranhão state, Brazil), between 1999 and 2016. The cohort included adults older than 19 years with a histopathological diagnosis of thyroid cancer. All patients had preoperative FNA biopsies of the malignant nodule available for analysis and were followed up for at least 1 year with biochemical (thyroglobulin and antithyroglobulin antibody) and imaging tests (ultrasound or radioiodine whole-body scan). After exclusion of patients not meeting these criteria, the final cohort comprised 153 patients.

The following information was collected from each patient: age, gender, Bethesda category of the preoperative FNA, size of the nodule, extent of surgical resection, histological variant, extrathyroidal extension, vascular invasion, margin status, presence of lymph node metastases and distant metastases, MR, RR, and the dynamic stratification on the last medical appointment.

All statistical analyses were performed using GraphPad Prism 5. Continuous and categorical variables are presented as mean ± standard deviation (SD). Fisher's exact test was used to analyze categorical variables. P values below 0.05 were considered statistically significant.

The institution's ethics committee approved the study.

## RESULTS

The final group comprised 153 patients with a mean age of 43.11 ± 12.8 years, of whom 96% (n = 147) were female. The most common tumor type was PTC (79.7%, n = 122) and the Bethesda II category was the most frequent FNA result (29.4%, n = 45). A total of 32.7% of the FNA samples yielded false-negative results. Table 1 shows the clinical characteristics of the entire cohort.

**Table 1:** Clinical characteristics of 153 patients with differentiated thyroid carcinoma

Characteristics		
Gender		
	Female	147 (96%)
	Male	6 (4%)
Age in years (mean ± SD, range)		43.11 ± 12.8 (20 – 75)
Multifocality		
	Yes	38 (24.8%)
	No	115 (75.2%)
Histology		
	Papillary	122 (79.7%)
	Follicular	26 (17%)
	Hürthle cell	5 (3.3%)
Bethesda category		
	I	5 (3.3%)
	II	45 (29.4%)
	III	17 (11.1%)
	IV	33 (21.6%)
	V	24 (15.7%)
	VI	29 (18.9%)

SD: standard deviation.

According to the TNM staging system, 90.8% (n = 139) of the patients were stage I, 3.9% (n = 6) were stage II, 4.6% (n = 7) were stage III, and 0.7% (n = 1) were stage IV. There was no relationship between the FNA result and the TNM staging system (p > 0.05).

Table 2 shows the results of the RR analysis. In total, 54.5% (n = 12 out of 22) of the high-risk patients had a Bethesda V or VI FNA result, while 31.3% (n = 41 out of 131) of low- and intermediate-risk patients had such results. However, no relationship was observed between the FNA result and the RR (Table 3). We were able to identify the dynamic stratification system of 133 of the 153 patients, since 20 patients were lost to follow-up. Our data showed that 83.4% (n = 111) of the patients had an excellent response, 8.3% (n = 11) had an indeterminate response, 5.3% (n = 7) had an incomplete biochemical response, and 3.0% (n = 4) had an incomplete structural response. Table 4 describes the relationship between the FNA results and an excellent response to treatment. Patients with a Bethesda II FNA result had better dynamic stratification classifications than patients with Bethesda III, IV, V, or VI FNA results (p < 0.05).

**Table 2:** Recurrence risk in 153 patients with differentiated thyroid carcinoma

Recurrence risk	N
Low	74 (48.4%)
Intermediary	57 (37.2%)
High	22 (14.4%)

**Table 3:** Relationship between Bethesda V or VI categories and a high recurrence risk (RR)

Bethesda V or VI	High RR	
	Yes	No
Yes	12 (25.5%)	35 (74.5%)
No	10 (9.4%)	96 (90.6%)

p = 0.051

**Table 4:** Relationship between fine-needle aspiration results and dynamic classification

	Excellent response		
	Yes	No	
Bethesda V and VI	35 (74.5%)	12 (25.5%)	p > 0.05
Bethesda III and IV	37 (84.1%)	7 (15.9%)	p > 0.05
Bethesda II	35 (92.1%)	3 (7.9%)	p < 0.05

There was no relationship between each Bethesda category and extrathyroidal extension (p > 0.05). However, when patients with a Bethesda V or VI result were analyzed as a single group, the group presented a relationship with the occurrence of extrathyroidal extension (p < 0.05). Also, patients with Bethesda V and VI results had more often vascular invasion than patients in other groups, and no relation was found between the FNA result and incomplete tumor resection (p > 0.05).

Finally, there was a progressive relationship between FNA results and lymph node metastasis, with Bethesda V and VI categories showing the highest risks.

## DISCUSSION

In our study, the patients had a mean age at diagnosis of 43.11 ± 12.8 years, and the cohort included women in a proportion above the one previously described in the literature (6). A possible reason for this may be the fact that women tend to seek medical care more frequently than men. As a result, men with DTC may be underdiagnosed or diagnosed at more advanced stages.

According to the histopathological DTC subtype, our cohort showed a distribution similar to that in previous studies, namely, PTC 79.7% *versus* 85%, respectively, and FTC 17% *versus* 12%, respectively (4).

At diagnosis, most of our patients (90.8%) were categorized as stage I, and only one patient was categorized as stage IV. This information is aligned with results from other studies in the literature (6) and shows that in most of our patients, the disease was identified at initial stages. We found no relationship between the FNA result and the TNM staging system (p > 0.05). The clinical course of malignant thyroid tumors varies widely. Most patients with DTC have a good prognosis when treated properly, and the mortality rates in these cases are similar to those in the general population (2). However, the percentage of patients who present recurrence is not negligible, while some fail to respond to conventional therapies and may even die of the disease. The TNM staging system is used to determine the disease-related mortality rate (7).

When we analyzed the relationship between the FNA results and the RRs, we observed that 54.5% of the high-risk patients had a Bethesda V or VI FNA result; however, this association was not statistically significant. VanderLaan and cols., in a study with 474 patients with preoperative FNA and diagnosed with PTC, observed that more patients with a Bethesda VI FNA result had a high RR (p < 0.05) (8).

We also observed an excellent response to treatment during follow-up in 74.5%, 84.1%, and 92.1% of the Bethesda V/VI, III/IV, and II patients, respectively. In fact, the Bethesda II group had a better dynamic staging (p < 0.05). This suggests that this group may receive a less aggressive treatment.

Our data also showed a positive relationship between Bethesda V and VI and extrathyroidal extension (p < 0.05), vascular invasion (p < 0.05), and lymph node metastasis (p < 0.05). On the other hand, there was no relationship between the Bethesda I, II, III, and IV categories and extrathyroidal extension and vascular invasion. Moreover, there was no relationship between the FNA result and incomplete tumor resection.

Liu and cols. analyzed 1,291 patients with PTC and described a progressive relationship between Bethesda III, IV, V, and VI FNA results and extrathyroidal extension, lymph node metastases, and incomplete tumor resection. The authors observed no relationship between these Bethesda categories with vascular invasion (9). VanderLaan and cols. also observed a higher incidence of lymph node metastases, vascular invasion, and extrathyroidal extension in Bethesda VI patients (8).

In a study including 360 patients with DTC, Kleiman and cols. found a strong relationship between the Bethesda V and VI categories with extrathyroidal extension and lymph node metastases (10).

Our data suggest that patients with a Bethesda V or VI FNA result may have more aggressive features related to a poor prognosis and, consequently, may receive a more aggressive treatment. Therefore, along with surgery, FNA results may help the treatment of patients with PTC.

Our study has some limitations. Among all patients with DTC included in the cohort, the Bethesda II was the most frequent category found in preoperative FNAs (29.4%). This is not aligned with the estimated MR in this Bethesda category (2) and may have influenced some of the results. Unfortunately, we were unable to identify other preoperative factors, such as suspicious ultrasound features, because several patients only started following up with our group after surgery.

In conclusion, the FNA results of thyroid nodules are helpful in guiding the initial treatment and may influence the follow-up of patients with DTC. Bethesda II patients show a better dynamic stratification, while Bethesda V or VI patients have an increased risk of extrathyroidal extension, vascular invasion, and lymph node metastasis. More studies are important to analyze how the FNA result impacts the treatment and follow-up of DTC.

## References

[1] Lamartina L, Grani G, Durante C, Borget I, Filetti S, Schlumberger M. Follow-up of differentiated thyroid cancer – what should (and what should not) be done. Nat Rev Endocrinol. 2018;14(9):538-51. 10.1038/s41574-018-0068-3 30069030

[2] Haugen BR, Alexander EK, Bible KC, Doherty GM, Mandel SJ, Nikiforov YE, et al. 2015 American Thyroid Association Management Guidelines for Adult Patients with Thyroid Nodules and Differentiated Thyroid Cancer: The American Thyroid Association Guidelines Task Force on Thyroid Nodules and Differentiated Thyroid Cancer. Thyroid. 2016;26(1):1-133. 10.1089/thy.2015.0020 PMC4739132 26462967

[3] Tuttle RM, Leboeuf R. Follow up approaches in thyroid cancer: a risk adapted paradigm. Endocrinol Metab Clin North Am. 2008;37(2):419-35. 10.1016/j.ecl.2008.02.008 18502335

[4] Coeli CM, Brito AS, Barbosa FS, Ribeiro MG, Sieiro APAV, Vaisman M. Incidência e mortalidade por câncer de tireóide no Brasil. Arq Bras Endocrinol Metab. 2005;49(4):503-9. 10.1590/s0004-27302005000400006 16358077

[5] Hegedüs L. The thyroid nodule. N Engl J Med. 2004;351(17):1764-71. 10.1056/NEJMcp031436 15496625

[6] Siegel RL, Miller KD, Jemal A. Cancer statistics, 2016. CA Cancer J Clin. 2016;66(1):7-30. 10.3322/caac.21332 26742998

[7] Rosário PW, Ward LS, Carvalho GA, Graf H, Maciel RMB, Maciel LMZ, et al. Nódulo tireoidiano e câncer diferenciado de tireoide: atualização do consenso brasileiro. Arq Bras Endocrinol Metab. 2013;57(4):240-64.

[8] VanderLaan PA, Marqusee E, Krane JF. Features Associated with Locoregional Spread of Papillary Carcinoma Correlate with Diagnostic Category in The Bethesda System for Reporting Thyroid Cytopathology. Cancer Cytopathol. 2012;120(4):245-53. 10.1002/cncy.21189 PMC3739973 22434789

[9] Liu X, Medici M, Kwong N, Angell TE, Marqusee E, Kim MI, et al. Bethesda categorization of thyroid nodule cytology and prediction of thyroid cancer type and prognosis. Thyroid. 2016;26(2):256-61. 10.1089/thy.2015.0376 PMC4754507 26563459

[10] Kleiman DA, Beninato T, Soni A, Shou Y, Zarnegar R, Fahey TJ. Does Bethesda category predict aggressive features in malignant thyroid nodules? Ann Surg Oncol. 2013;20(11):3484-90. 10.1245/s10434-013-3076-5 PMC4025924 23812773

